# The Mechanisms of Virulence Regulation by Small Noncoding RNAs in Low GC Gram-Positive Pathogens

**DOI:** 10.3390/ijms161226194

**Published:** 2015-12-14

**Authors:** Stephanie Pitman, Kyu Hong Cho

**Affiliations:** Department of Biology, Indiana State University, Terre Haute, IN 47809, USA; spitman@sycamores.indstate.edu

**Keywords:** small noncoding RNAs, regulatory RNAs, virulence control mechanism, Gram (+) pathogens, CU interaction motifs, two-component regulators, riboswitches, quorum sensing, toxin/antitoxin systems

## Abstract

The discovery of small noncoding regulatory RNAs (sRNAs) in bacteria has grown tremendously recently, giving new insights into gene regulation. The implementation of computational analysis and RNA sequencing has provided new tools to discover and analyze potential sRNAs. Small regulatory RNAs that act by base-pairing to target mRNAs have been found to be ubiquitous and are the most abundant class of post-transcriptional regulators in bacteria. The majority of sRNA studies has been limited to *E. coli* and other gram-negative bacteria. However, examples of sRNAs in gram-positive bacteria are still plentiful although the detailed gene regulation mechanisms behind them are not as well understood. Strict virulence control is critical for a pathogen’s survival and many sRNAs have been found to be involved in that process. This review outlines the targets and currently known mechanisms of *trans*-acting sRNAs involved in virulence regulation in various gram-positive pathogens. In addition, their shared characteristics such as CU interaction motifs, the role of Hfq, and involvement in two-component regulators, riboswitches, quorum sensing, or toxin/antitoxin systems are described.

## 1. Introduction

In order for pathogens to successfully infect and colonize a host, strict control of virulence is critical. Recently, a group of noncoding RNAs called small RNAs (sRNAs) has been recognized as an essential factor of virulence control in many pathogens. These bacterial sRNAs have become the most abundant class of post-transcriptional regulators [1].

Small RNAs can be classified by the targets they regulate. Those co-transcribed with their regulatory targets (riboswitches) or bound to the complementary mRNA with perfect base pairing (antisense regulatory RNAs) are classified as *cis*-acting sRNAs. On the other hand, sRNAs that regulate different RNA, DNA, or proteins are designated as *trans*-acting sRNAs [2]. This review is limited to mostly *trans*-acting sRNAs with RNA targets (for a review of *cis*-acting gram-positive antisense sRNAs, see [3]).

The functions of *trans*-acting sRNAs are to regulate translation initiation, RNA stability, or protein activity. The role of most sRNAs is to control translation. This control occurs primarily by one of three different ways: (1) The sRNA directly binds to and blocks the ribosome-binding site (RBS) to inhibit translation; (2) the sRNA binds to induce structural changes of the RBS to inhibit or promote translation; or (3) the sRNA binds in order to block a ribosome standby site to promote translation [1].

Computational analysis has been widely used for the discovery of sRNAs in various bacteria. Sophisticated algorithms are often used to identify sequences as potential sRNAs [4,5]. However, the use of computational analysis for identifying potential sRNAs is often difficult because the identified sequences are very small and usually do not possess nucleotide motifs. In addition, sRNAs tend to be conserved only in highly related bacterial species and/or only expressed in “pathotype-specific” strains of the same species [4]. Hence, computational analysis alone is not enough to confirm a potential sRNA. Common methods to test the presence of sRNA include Northern blotting, reverse transcriptase real-time PCR, and RNA sequencing [5,6,7,8].

Many studies have been performed on sRNAs present in gram-negative bacteria, especially *E. coli*. The sRNAs discovered in the early days were on plasmids and transposons in *E. coli*. *E. coli* is also a well-characterized model organism with diverse genetic tools, which makes it ideal for sRNA research. Gram-positive bacteria have only recently been given greater attention in the search for sRNAs. Virulence control is a critical aspect of pathogenesis and studying the role of sRNA involvement in the virulence control will lead to insights into pathogenesis as a whole. The aim of this review is to provide a comprehensive overview of the known and speculated regulatory mechanisms by *trans*-acting sRNAs controlling virulence in gram-positive pathogens (Table 1). Those sRNAs in each pathogen are described first, and then their mechanisms of gene regulation and shared characteristics are delineated.

**Table 1:** Small regulatory RNAs influencing virulence of gram-positive pathogens.

Bacteria	Small RNA	Target mRNA	Protein Encoded in Target mRNA	References
*S. pyogenes*	PelRNA	*speB*	Cysteine protease	[1]
		*emm*	M protein	
		*sic*	Streptococcal inhibitor of complement	
		*nga*	NAD-glycohydrolase	
	FasX	*ska*	Streptokinase	[9,10]
		*sagS*	Streptolysin S	
		*fbp54*	Fibronectin binding protein	
		*mrp*	M-related protein	
	RivX	*mga*	Virulence regulator	[11]
*S. pneumoniae*	F20	Not known	–	[12]
	F32	*–*	F32 encodes a tmRNA	[12]
*S. aureus*	RNAIII	*hla*	α-hemolysin	[1,2,13]
		*rot*	Repressor of toxins	[14,15,16]
		*sa1000*	Fibrinogen-binding protein	[1,15]
		*spa*	Protein A	[17]
		*coa*	Coagulase	[18]
		*lytM*	Peptidoglycan hydrolase	[19]
		*map*	MHCII analogous protein	[20,21]
	SprD	*sbi*	Immunoglobulin-binding protein	[22]
	Psm-mec	*agrA*	Virulence regulator	[23]
	ArtR	*sarT*	Transcriptional regulator of *hla*	[24]
*L. monocytogenes*	SreA	*prfA*	Virulence master regulator	[25]
		*agrD*	Precusor of autoinducer	
	SreB	*prfA*	Virulence master regulator	[25]
	LhrA	*chiA*	Chitinase	[26]
	LhrC	*lapB*	Virulence adhesin	[6,27]
*C. perfringens*	VR-RNA	*colA*	Collagenase	[28,29,30,31]
		*pfoA*	Pore-forming toxin Perfringolysin A	
		*vrr*	VirRS-regulated sRNA	
		*virT*	The VirT sRNA	
		*virU*	The VirU sRNA	
		*ccp*	Cysteine protease α-clostripain	
	VirX	*pfoA*	Pore-forming toxin Perfringolysin A	[31]
		*plc*	Alpha-toxin phospholipase C	
		*colA*	Collagenase	
	VirU	*vrr*	VirRS-regulated sRNA	[31]
		*pfoA*	Pore-forming toxin Perfringolysin A	
		*virT*	The VirT sRNA	
	VirT	*pfoA*	Pore-forming toxin Perfringolysin A	[31]
		*colA*	Collagenase	
*E. faecalis*	Ef0408-0409	Fst	Peptide toxin	[32]

### 1.1. Streptococcus pyogenes

*Streptococcus pyogenes* (*S. pyogenes*), commonly referred to as Group A *Streptococcus* (GAS), is a human pathogen capable of inducing a wide range of diseases including pharyngitis, impetigo, toxic shock-like syndrome, and necrotizing fasciitis. To this point, only three sRNAs, FasX, RivX, and Pel, have been tied to the regulation of virulence gene expression in GAS [9,11,33,34]. Recent efforts have been made to identify other sRNAs in *S. pyogenes*, and an additional 40 sRNAs have been discovered [5,35,36,37]. However, putative targets and involvement in virulence control of these sRNAs have yet to be experimentally determined.

The fibronectin/fibrinogen binding/hemolytic activity/streptokinase regulator X (FasX) has been shown to regulate five separate virulence genes [9]. The mechanisms behind FasX and target binding have been studied only for two of the virulence factors, thrombolytic agent streptokinase and the pilus [9,10,38]. Streptokinase, a secreted virulence factor, promotes conversion of the protein plasminogen into protease plasmin [39]. This benefits the bacteria by degrading tissue barriers and blood clots [40]. FasX interacts with the mRNA of the streptokinase gene, *ska*, and this FasX:*ska* mRNA interaction increases the mRNA stability resulting in a 10-fold increase in *ska* mRNA abundance [10]. FasX contributes to *ska* mRNA stability through the creation of a 5ʹ-end secondary structure and must remain bound to the *ska* mRNA in order to maintain the increased stability [41].

The second virulence factor confirmed to be FasX-regulated is the pilus [38]. The pili are important for virulence by allowing the pathogen to adhere to host cells and begin colonization. FasX base pairs to the mRNA of *cpa*, the first gene in the pilus biosynthesis operon, and encodes a collagen-binding pilus protein [42]. Contrary to FasX:*ska* mRNA interaction, the FasX:*cpa* mRNA interaction negatively regulates target mRNA expression [2]. This negative regulation is achieved through the inhibition of *cpa* mRNA translation by reducing the availability of ribosomes to the *cpa* mRNA ribosome binding site (RBS) [38]. FasX employs CU-rich complementary sequences at binding sites [2]. The FasX complementary sequences for *ska* and *cpa* mRNAs are 5ʹ-UCAAUCCCC and 5ʹ-UUGUUUUCUCUCUCUC, respectively [2].

The FasX sRNA also regulates the expression of *sagS* that encodes streptolysin S, and *fbp54* and *mrp* encoding fibronectin-binding proteins. The mechanism for these regulations has not yet been discovered [1].

The discovery of the RivX sRNA was significant because it tied together the CovR regulatory cascade and the Mga regulon [11]. The two-component system CovR/CovS and the Mga regulator are the best-studied virulence gene regulators in *S. pyogenes* [11]. The two component system CovR/CovS influences transcription of 15% of all chromosomal genes in *S. pyogenes* and represses the expression of major virulence factors such as the capsule, streptokinase, streptolysin S and streptodornase [43]. Mga is an activator critical for the expression of many virulence genes such as *emm* (M protein), *scpA* (streptococcal C5A peptidase), *sic* (streptococcal inhibitor of complement-mediated lysis), and *fba* (fibronectin-binding protein A) [11]. Neither direct targets of RivX nor the mechanism behind the sRNA has been identified, although it has been suggested that RivX either enhances expression of the Mga protein or works indirectly through another unknown regulatory factor [11].

Pel (pleiotropic effect locus) is a bifunctional sRNA able to both encode for a protein and have regulatory properties [44]. Pel was reported to control the following virulence factors: the hemolysin streptolysin S (SLS), M protein, streptococcal inhibitor of complement (Sic), and streptococcal cysteine protease (SpeB) [33,34]. The mechanism by which Pel controls the expression of virulence factors is currently unknown and no direct targets have been discovered. The untranslated regions (UTRs) of Pel are predicted to act in *trans* on target mRNAs. It is also possible that Pel could work in conjunction with a protein co-factor [44]. However, the existence of the Pel sRNA has been questioned because some studies were unable to replicate previous results [36,45].

### 1.2. Streptococcus pneumoniae

*Streptococcus pneumoniae* (*S. pneumoniae*) is part of the normal human microflora and is able to asymptomatically colonize the upper respiratory tract in children and healthy adults. The transition to an opportunistic pathogen causes many human diseases varying in severity from otitis media to sepsis, meningitis, sinusitis or endocarditis. The processes that trigger this transition after the initiation of the respiratory tract infection are currently unknown [46]. The importance of sRNAs in the regulation of virulence in *S. pneumoniae* was recognized recently through the use of targeted genetic knockouts and Tn-seq transposon mutagenesis screening [12]. Despite the research, however, the targets and mechanisms by which sRNAs affect virulence have yet to be elucidated (for a list of studied small RNAs in *S. pneumoniae* not known to be involved in virulence, see [46]).

To date, two sRNAs have been discovered to play a role in virulence. Deletion of these sRNAs, F20 and F32, resulted in significant deficiency in the adhesion and invasion to nasopharyngeal or endothelial cells, respectively [12]. Interestingly, F32 encodes a tmRNA, some of which have been suggested to affect the pathogenesis of other bacteria [12]. tmRNA has the properties of both tRNA and mRNA and forms a complex with ribosomal protein S1, elongation factor Tu, and Smp. This ribonucleoprotein complex facilitates the elongation process of ribosomes that have stalled in the middle of protein biosynthesis. Pleiotropic effects were exhibited in the F20 sRNA mutation. The decrease of purine metabolism proteins and the increase of proteins in DNA synthesis and repair pathways together in the F20 mutant imply that F20 plays a critical role in DNA metabolism [12]. Future studies will be necessary to determine the targets and underlying mechanisms of the sRNAs and the roles they play in virulence regulation.

The two-component system CiaRH has also been implicated in virulence control in *S. pneumoniae* as well as other streptococcal strains [47,48,49,50,51]. Of the 15 promoters regulated by CiaRH, five express sRNAs [52]. Besides virulence, CiaRH itself is involved in the regulation of many processes including competence development, β-lactam resistance, and lytic processes [50,51]. Investigation into the involvement of the CiaRH-regulated sRNAs in virulence has not yet been pursued.

### 1.3. Staphylococcus aureus

*Staphylococcus aureus* (*S. aureus*) has become a pathogen of major concern due to its ability to rapidly acquire antibiotic resistance and cause both nosocomial and community-associated infections. *S. aureus* is a commensal pathogen found in 30%–70% of the population but only becomes a health threat upon entering a weak point in the skin [22]. At that point, the pathogen can induce a multitude of diseases ranging from skin infections to life-threatening systemic infections [53].

The most studied sRNA thus far in *S. aureus* is RNAIII, which is the effector of the *agr* system used for quorum sensing. The *agr* system has two transcription units, RNAII and RNAIII. After reaching a certain threshold of cell density, AgrA, the response regulator of the *agr* system, activates the transcription of its own operon and subsequently the transcription of RNAIII [14]. RNAIII activates translation of the extracellular virulence factor gene *hla*, which encodes alpha-hemolysin. RNAIII is also an inhibitory sRNA for the transcripts of many surface virulence factor genes such as *rot* (pleiotropic transcriptional factor), *sa1000* (fibrinogen-binding protein), *sa2261* (ABC transporter), *spa* (surface adhesion protein A), *lytM* (peptidoglycan hydrolase), *map* (major histocompatibility complex class II analogous protein), and *coa* (staphylocoagulase) [1].

RNAIII is known to activate translation of alpha-hemolysin. This activation occurs through competitive binding of 3′-RNAIII that prevents the formation of an inhibitory secondary structure in the *hla* mRNA and allows the *hla* RBS to become available [2,13].

Map is an important adhesion mediator between *S. aureus* infection and the host’s immune system and is suggested to play a role in septic shock and fever [19]. RNAIII up-regulates the expression of MAP by base-pairing to *map* mRNA, most likely using hairpin 4 of RNAIII [20].

The Rot protein is a transcriptional regulatory protein involved in virulence gene expression [14]. RNAIII inhibits the translation of *rot* mRNA through base pairing. RNAIII contains 14 separate hairpin structures used for binding to various targets [54].Three hairpin domains of RNAIII at the 3′-end, hairpins 7, 13, and 14 are utilized to bind to three UUGGGA motifs in *rot.* Preference is given to hairpin 14 for binding to the Shine-Dalgano (SD) sequence of the *rot* mRNA [15].

The *sa1000* gene encodes the fibrinogen-binding protein that is highly conserved in all *S. aureus* strains [15]. The *sa1000* mRNA contains two long hairpin structures, I and II. RNAIII hairpin 13 binds to *sa1000* mRNA hairpins I and II to induce structural changes that cover the RBS of the *sa1000* mRNA. Without hairpin 13, this interaction cannot occur [15]. The formation of this duplex prevents ribosomal binding, thus the translation of the *sa1000* mRNA is inhibited [15].

Surface protein A, encoded by the *spa* gene, is a major *S. aureus* virulence factor [16]. RNAIII inhibits translation of the *spa* mRNA through a loop–loop interaction. The hairpin 13 at the 3′-end of RNAIII anneals through complementary base pairing to the 5′ end of the *spa* mRNA. In vitro, this complex alone is able to inhibit the formation of the translation initiation complex. However, *in vivo*, RNase III is also required to degrade the mRNA and permanently stop translation [16].

The *coa* gene encodes an extracellular protein staphylocoagulase*.* After forming a complex with prothrombin, staphylocoagulase promotes fibrin formation in human plasma, contributing to bacterial camouflage against the immune system [17]. Specificity of RNAIII is acquired by the addition of a secondary loop–loop interaction. The duplex formed between *coa* mRNA and RNAIII comprises a loop–loop interaction in the *coa* ORF that induces changes surrounding the RBS and masks the SD sequence. Comprised of two consecutive areas of 13 base-pairings separated by an internal loop, this imperfect duplex prevents *coa* translation [17].

The *lytM* gene encodes an autolysin and RNAIII interacts with the *lytM* mRNA 5′-UTR. This binding results in the blocking of the RBS [18].

Recently, the sRNA ArtR was discovered to regulate alpha-toxin expression through the target mRNA *sarT*. After binding to the 5′-UTR of *sarT*, ArtR induces degradation of *sarT* mRNA by RNaseIII. This in turn promotes alpha-toxin expression by activating the expression of *hla* through an indirect pathway [21].

Another recently discovered sRNA in *S. aureus* is the Psm-mec dual function sRNA. The *psm-mec* gene confers methicillin resistance to MRSA strains through the cytolytic toxin PSMα. However, Psm-mec also inhibits the translation of *agrA* mRNA through 5′-end base pairing [24]. Further studies are needed to understand the complete mechanism.

### 1.4. Listeria monocytogenes

*Listeria monocytogenes* (*L*. *monocytogenes*) is a food-borne pathogen and is the cause of listeriosis, a rare but severe human infection with an overall 30% mortality rate. *L. monocytogenes* is a unique pathogen due to its ability to infect and multiply inside of host cell macrophages. A recent study suggests that sRNAs are involved in macrophage infection [55]. However, no target mRNAs or mechanisms for intracellular-expressed sRNAs have been identified.

Many different sequencing tools have been used to search for sRNAs in *L. monocytogenes* [55,56,57]. The SOLiD HTS platform was recently employed in a search for new sRNAs and was able to determine 9 novel sRNAs in *L. monocytogenes* [8]. Northern blot analysis and qRT-PCR supported the results of computational analysis, but neither their targets nor mechanisms have been further investigated [8].

To date, only a few targets of *L. monocytogenes* sRNAs have been identified [1]. Currently, the only sRNAs in *L. monocytogenes* known to be involved in virulence control are SreA, SreB, LhrA, and LhrC [6].

Some riboswitches in *Listeria* have the unique ability to also act as sRNAs. SreA and SreB have been described as S-adenosylmethionine (SAM) riboswitches. Riboswitches are gene expression regulatory structures that form in mRNA. Unique from other RNA regulatory structures, riboswitches are bound by small ligands to control downstream genes. Found only in bacteria, they are thought to be one of the oldest types of regulatory systems [58]. Interestingly, SreA and SreB can also act in *trans* as noncoding regulatory RNAs in *L. monocytogenes* [25]. SreA and SreB together control PrfA, a master virulence regulator, through binding to the 5′-UTR of the *prfA* mRNA [25]. It is unknown whether other riboswitches have the capacity to act as small RNAs as well, but after finding the duality of SreA and SreB, researchers are searching for more.

SreA has two known target mRNAs, *prfA* and *agrD* (*lmo0049*), which regulate virulence and quorum sensing, respectively [1,25]. When SreA is not present, PrfA expression increases whereas the expression of AgrD decreases. PrfA does not control AgrD expression, so the regulation of the expression of PrfA and AgrD by SreA seems to be independent [56]. SreA is thought to interact directly with the *prfA-*UTR because of its high degree of complementarity between paired region 3 and the distal side of the *prfA-*UTR. In addition, base substitution mutations in either SreA or *prfA-*UTR destabilize the interaction [25]. PrfA is also controlled by a thermosensor that inhibits PrfA expression below temperatures permissive for infection [7]. SreA activity is also dependent on the thermosensor, so SreA is unable to interact with the thermosensor at lower temperatures [25]. SreB binds to *prfA* as well and controls its expression, but little more is known of this interaction [25].

The other *Listeria* sRNAs involved in virulence are LhrA and LhrC. LhrA is encoded by the gene *lmo2257*, which has not been found to be homologous to any known genes [27]. To date, LhrA is the only known example of sRNA in gram-positive bacteria that requires the assistance of the Hfq protein [59]. Hfq-dependent sRNAs usually bind to the 5′-region of the target mRNA, which represses translation initiation and stimulates degradation of the mRNA [26]. LhrA has a negative effect on the chitinolytic activity of *L. monocytogenes* [26]. Extensive complementarity exists between the translation initiation region of the 5′-end of the *chiA* mRNA and LhrA. Hfq is necessary for the formation of the LhrA-*chiA* mRNA duplex [26].

LhrC, encoded in the intergenic region (IGR) between *cysK* and *sul*, is highly conserved among *Listeria* species and is expressed in five almost identical gene copies. Its expression is regulated by the two-component system LisRK [6]. The exact role of LhrC is currently unknown. A target is the *lapB* mRNA which encodes a cell-wall anchored adhesion protein involved in virulence and is a target for all five sRNA copies [6]. The LhrC:*lapB* mRNA interaction was different from what was predicted. The sRNA and mRNA have two complementary sequences that were the predicted binding targets. However, LhrC has three redundant CU-rich motifs (5′-UCCC) and each of these CU-rich motifs is able to recognize the target RNA [6]. Mutations in the single-stranded portion of the LhrC sRNA did not affect its binding to *lapB*. However, mutations in either loop A or the terminator loop resulted in decreased interaction between the RNAs. Lastly, if the binding regions of both loops are mutated, the interaction of LhrC:*lapB* mRNA is almost nonexistent [6].

### 1.5. Clostridium perfringens

*Clostridium perfringens* (*C. perfringens*) is a spore-forming bacterium pervasive throughout soil and sewage as well as animal intestinal tracts. Most known for its role in food poisoning, *C. perfringens* can also be the cause of many other diseases including gas gangrene, necrotic enterititis, and clostridial myonecrosis. Which disease develops is dependent on toxins produced by the particular strain [60].

Only two sRNAs have been well studied in *C. perfringens*: the sRNA VR-RNA (VirR-regulated RNA) that is part of the VirR/VirS two-component system that controls toxin production [61] and VirX which works independently of the VirR/VirS system and represses sporulation regulation [62]. The other known sRNAs in *C. perfringens* are VirU and VirT which are both also involved in the regulation of various toxins [60]. Understanding the mechanisms behind the sRNAs in all species of pathogenic clostridia has been limited due to the difficulty of genetic manipulation of the bacteria [60].

VR-RNA is a part of the VirR/VirS two-component system that is an important regulator for gene expression in *C. perfringens* [28,61]. VR-RNA, a 386 nt sRNA, is positively regulated by VirR/VirS and regulates the expression of various toxins and housekeeping genes [28,29,63,64]. Most notably, VR-RNA controls the expression of the membrane active toxin *plc* (α toxin) and *colA* (κ toxin, collagenase) genes. The 3′-region of VR-RNA plays a key role in the regulation of both *plc* and *colA* genes [29]. The 5′-UTR of the *colA* mRNA binds to a highly complementary 3′-region of VR-RNA for greater *colA* mRNA stability [28]. It has been hypothesized that the binding of VR-RNA to the *colA* mRNA could regulate expression by preventing the formation of a stem-loop structure in the *colA* mRNA [30]. Although a few target genes have been identified, the mechanism behind VR-RNA regulation is still not well understood [28,30].

Unlike VR-RNA, VirX has been shown to regulate the *pfoA*, *plc*, and *colA* mRNA independently of the VirR/VirS system [31]. A recent study suggests that VirX also regulates the expression of enterotoxin production in *C. perfringens* [62]. The sRNA VirU has not been well studied yet, but was found to act as either a positive regulator or as a stabilizer for *vrr*, *pfoA*, and *virT* [61]. The same study discovered that the sRNA VirT negatively regulates *pfoA* and *colA* transcription [61]. The molecular mechanisms of gene regulation by the sRNAs VirX, VirU, and VirT have not yet been studied.

### 1.6. Enterococcus faecalis

*Enterococcus faecalis* (*E. faecalis*) is a normal human and animal microflora residing in the gastrointestinal tract. The mechanisms that cause *E. faecalis* to become pathogenic are not well understood [65,66]. The young and immunologically compromised individuals are most likely to be affected by the pathogen [65]. Possible infections include meningitis, pneumonia, endocarditis, and most notably, catheter-associated urinary tract infections [65,66]. A few transcriptional regulators have been found to be involved in virulence [67,68]. *E. faecalis* does not possess the normal virulence factors such as proinflammatory toxins. Rather, it has “opportunism factors”, which allow it to survive hostile conditions as introduced in a stressful environment [66]. Thus far, the search for sRNAs influencing virulence of *E. faecalis* has not been attempted.

Homologous to the RNAII portion of the toxin–antitoxin (TA) system, sRNA *ef0408-0409* has been studied as a virulence-affecting sRNA in *E. faecalis* [32,66,69,70]*.* Typically, both the toxin and the antitoxin are proteins. In *E. faecalis*, however, the translation of toxin mRNA is repressed by the antitoxin sRNA *ef0408-0409* [70]. The target of the antitoxin sRNA is Fst, the peptide toxin [32]. Deletion of the *ef0408-0409* sRNA exhibited “hypervirulence” in *Galleria mellonella* worm models thereby supporting that *ef0408-0409* is a virulence-regulating sRNA. The mechanism of the virulence control by *ef0408-0409* has not yet been studied. The unexpected ability to delete the antitoxin sRNA *ef0408-0409* implies that the mechanism could be complex [66,70].

## 2. Common Features Shared by sRNAs in Gram-Positive Pathogens

Despite the differences in bacterial species and sRNA functions, many similarities exist when gram-positive sRNAs are compared as a whole. By analyzing these similarities, we can better understand the characteristics and regulation mechanisms of sRNAs. In this review, activation and repression mechanisms, CU interaction motifs, involvement in two-component regulators, riboswitches, quorum sensing, or toxin/antitoxin systems, and the role of Hfq are employed to describe gram-positive sRNA characteristics.

### 2.1. Regulation Mechanisms by Small RNAs: Activation or Repression of Gene Expression

**Figure 1:**
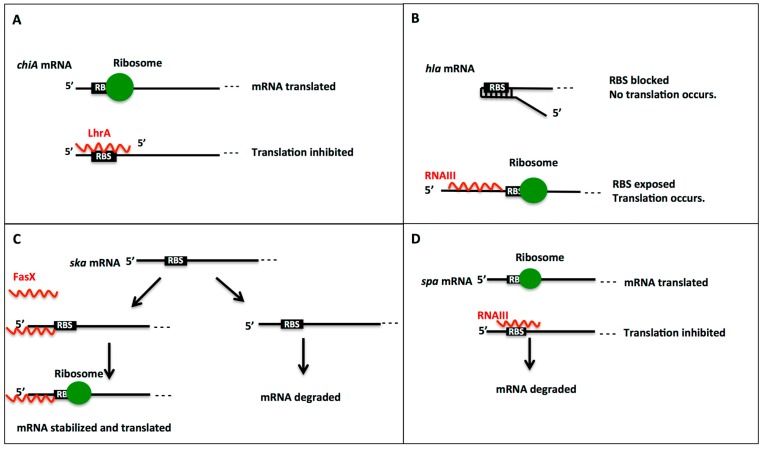
Regulation mechanisms of gene expression by sRNAs. (**A**) Translation repression by sRNA-binding. Small RNA LhrA regulates chitinolytic activity in *L. monocytogenes* through binding to *chiA* mRNA. LhrA binding negatively regulates chitinolytic activity through binding to the RBS and inhibiting translation. Extensive complementarity exists between the 3′-end of LhrA and the 5′-end of *chiA* mRNA. Without LhrA bound to the *chiA* mRNA, the ribosome can bind the RBS and translate the mRNA; (**B**) Translation activation by sRNA-binding. The *S. aureus hla* encodes extracellular α-hemolysin. When RNAIII is not bound to the *hla* mRNA, the mRNA conformation blocks the RBS and translation cannot occur. After RNAIII binds to the *hla* mRNA through a loop–loop interaction, the RBS is exposed and translation occurs; (**C**) Stabilization of mRNA by sRNA-binding. The most studied small RNA in *S. pyogenes*, FasX, positively regulates streptokinase through increasing the stability of the *ska* transcript. The binding of FasX to the 5′-end of the *ska* mRNA transcript protects the mRNA from degradation, so increases the abundance of streptokinase. Without the binding of FasX, the *ska* mRNA is degraded by RNases; (**D**) Promotion of mRNA degradation by sRNA-binding. An increase in target mRNA degradation occurs after the binding of RNAIII to the *spa* mRNA in *S. aureus*. Without RNAIII bound to the *spa* mRNA, the ribosome can bind and translation can proceed. When RNAIII binds, the loop–loop interaction between hairpin 13 of RNAIII and the *spa* mRNA complex not only inhibits the translation but also promotes the degradation of the *spa* mRNA in order to permanently stop translation.

Small RNAs can affect gene expression through activation or repression or in some cases both depending on target mRNAs (Figure 1). One such sRNA, RNAIII in *S. aureus* activates expression of *hla* and *map* but represses translation of all other known target mRNAs of *rot*, *SA1000*, *spa*, *lytM*, and *coa*.

Commonly, sRNAs repress gene expression by inhibiting target mRNA translation (Figure 1A). For example, RNAIII inhibits expression of the mRNAs of *rot*, *SA1000*, *spa*, *lytM*, and *coa* through base-pairing its 3′-hairpin loops with the target mRNAs and/or the use of RNAIII to degrade them [16]. For *rot* and *coa* mRNAs, inhibition occurs through the addition of a second loop–loop interaction which masks the SD sequence [1]. One of the most recently discovered sRNAs in *S. aureus*, Psm-mec, was shown to inhibit translation of *agrA* mRNA through base pairing with the 5′-end to the coding sequence of the target mRNA [1]. This sRNA:target mRNA interaction furthermore decreases the half-life of *agrA* [24]*.* Another example is the VirU sRNA in *C. perfringens*, which has been found to negatively regulate transcription of the mRNAs of *pfoA* and *colA.* An interesting gram-positive target mRNA-repressing sRNA is LhrA in *L. monocytogenes.* Currently, it is the only known gram-positive sRNA that requires the Hfq protein in order to function [27]. It is therefore not surprising that no LhrA homologs were identified in other bacterial species [27].

In some cases, sRNAs activate transcription of target mRNAs. This activation typically involves sRNA-binding to cause a conformational shift that exposes the RBS, as in the case of the RNAIII and *hla* mRNA interaction (Figure 1B). A loop–loop interaction occurs between the *hla* mRNA and RNAIII. The initial touching of the complementary regions on the loops is quickly followed by the binding of the straight region [2]. In addition, the formation of the VR-RNA:*colA* mRNA complex in *C. perfringens* exposes the RBS, allowing mRNA translation [28]. The VirU sRNA in *C. perfringens* also plays a role in activating the expression of its target mRNAs of *vrr*, *pfoA*, and *virT* although the mechanism is not yet understood [61]. VirX, another *C. perfringens* sRNA, also activates the expression of the *plc*, *colA*, and *pfoA* genes through unknown mechanisms [62].

The stability of target RNAs can also be affected by sRNA-binding. An example of increased stability is seen in the interaction between FasX and *ska* mRNA through the formation of a 5′-secondary loop structure on the mRNA (Figure 1C). Without FasX bound to the *ska* transcript, the mRNA is degraded, so the interaction must be maintained in order for translation to proceed [2].

A decrease in the stability of the mRNA transcript resulting in mRNA degradation can also be induced by sRNA-binding (Figure 1D). One such example is the RNAIII:*spa* mRNA interaction in *S. aureus*. The 3′ domain of RNAIII is sufficient to repress *spa* translation. However, this interaction forms a complex of double-stranded RNA, which is then specifically cleaved by RNase III in both the duplex and hairpin II of the *spa* mRNA. This cleavage allows other endo- or exoribonucleases to then rapidly degrade the *spa* mRNA [16]. In order to completely repress the expression of *spa*, the mRNA transcript must be degraded.

### 2.2. CU Repeats in sRNA:Target mRNA Interactions

**Figure 2:**
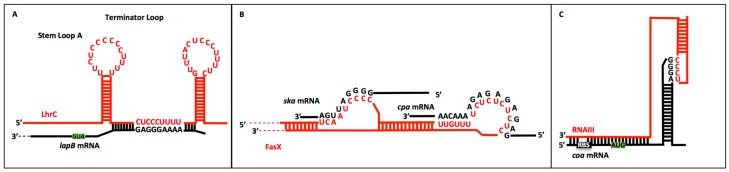
CU-rich motifs in the binding sites of sRNA to target mRNA appear to be conserved in gram-positive bacteria. Small RNAs are designated as red and target mRNAs are designated as black. The bolded nucleotides highlight the CU-rich motifs involved in sRNA:target RNA binding. (**A**) Predicted model of LhrC:*lapB* mRNA interaction in *L. monocytogenes.* An initial “kissing” interaction occurs between the loop structures of LhrC and the *lapB* mRNA through CU-rich motifs to increase specificity and is followed by straight region binding. This binding inhibits further transcription of the *lapB* gene by inducing the formation of a transcription terminator in the nascent *lapB* mRNA. Mutations of the CU-rich motif in either loop greatly decrease interaction between the RNAs, indicating the CU-rich motifs are necessary for target recognition; (**B**) Predicted model of FasX:target mRNA interaction in *S. pyogenes.* FasX utilizes two separate regions of conserved CU motifs for the binding to target mRNAs *ska (*streptokinase) and *cpa* (pilus)*.* The FasX:*ska* mRNA interaction creates a 10-fold increase in mRNA abundance and positively regulates gene expression as long as FasX remains bound. Through another loop structure in FasX, it also regulates expression of the *cpa* mRNA by reducing ribosome availability to the transcript; (**C**) Predicted model of RNAIII:*coa* mRNA interaction in *S. aureus.* The binding between RNAIII and the *coa* mRNA via a loop–loop interaction induces structural changes surrounding the ribosome binding site. These changes mask the ribosome binding site and prevent *coa* translation. 5′-AUG in the green background (Figure 2A,C) represents the start codon, and RBS in the black background (Figure 2C) indicates the ribosome binding site.

Sequences consisting of varying lengths and patterns have arisen as motifs in sRNA:mRNA interactions. Originally, it was theorized that UC nucleotide concentrations were important in binding for *S. aureus* sRNAs, specifically the UCCC sequence motif [71]. Since then, similar motifs have been discovered in other gram-positive pathogens (Table 2) (Figure 2). In *L. monocytogenes*, the LhrC sRNA contains three redundant CU-rich motifs, CUCCC, in the loop for target recognition, which together provide perfect complementarity with the *lapB* target mRNA [6]. In the *S. pneumoniae* pathogen, many *cia*-dependent small RNAs (csRNAs) contain a conserved CCUCCU motif that has been implicated in RBS blocking and thereby translation inhibition [37,52]. In *S. pyogenes*, the FasX sRNA binds to the target mRNAs, *ska* and *cpa*, using complementary and particularly UC-rich regions, UCAAUCCCC and UUGUUUUCUCUCUCUC, respectively [2]. Because the study of sRNAs in gram-positive bacteria is still relatively new, it is expected that more CU-rich binding motifs will appear.

**Table 2:** CU motifs in sRNA:mRNA interactions in gram-positive bacteria.

Bacteria	Small RNA	CU Motif	Target mRNA	Reference
*S. pyogenes*	FasX	5′-UCAAUCCCC	*ska*	[2]
		5′-UUGUUUUCUCUCUCUC	*cpa*	[2]
*S. pneumoniae*	csRNAs	5′-CCUCCU	Unknown	[52]
*S. aureus*	RNAIII	5′-UCCC	*coa*	[17,71]
*L. monocytogenes*	LhrC	5′-CUCCC; 5′-UUUU	*lapB*	[6]

### 2.3. Small RNAs as Effector Molecules of Two-Component Systems

**Figure 3:**
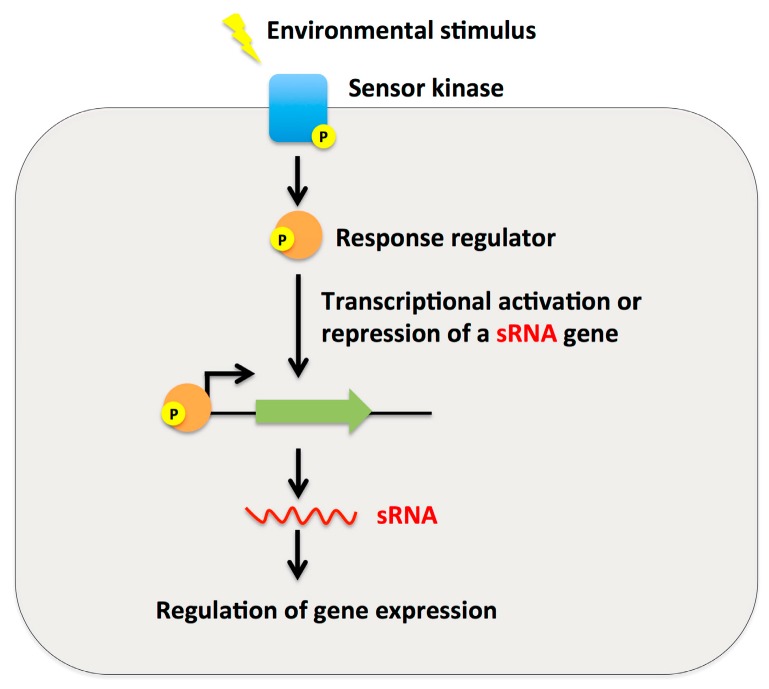
Small RNAs can be used as effector molecules of two component systems (TCS). Two component systems (TCS) allow bacteria to react to an environmental stimulus through activating or repressing gene transcription. The sensor kinase in the system receives an environmental signal and then becomes phosphorylated. This phosphate is then transferred to the response regulator, which binds near the promoter region to activate or repress transcription. In some cases, sRNAs work as effectors of two component systems. In *L. monocytogenes*, the TCS LisRK activates sRNA LhrC1-5 transcription. LhrC then goes on to affect the production of LapB, a cell wall adhesion protein. A similar example is seen in *C. perfringens* where the VirR component of the VirRS TCS is phosphorylated and activates transcription of the *vrr* gene, encoding for the small RNA VR-RNA. VR-RNA then proceeds to regulate the expression of the toxin gene *plc* and the collagenase gene *colA.* The CovRS TCS in *S. pyogenes*, in contrast, represses the expression of *rivX*, an sRNA with currently unknown targets and mechanism.

A two-component system (TCS) is a basic coupling mechanism that allows organisms to sense and respond to changes in environmental conditions. Highly sophisticated in design, they all vary to meet the needs of the particular signal circuit. The standard TCS consists of a histidine kinase in the membrane that monitors the environment, and a cytoplasmic response regulator that allows the cell to respond [72]. Two-component systems not only control gene expression directly but also indirectly through regulators such as sRNAs (Figure 3). A few gram-positive sRNAs are involved in such systems of regulation. RivX in *S. pyogenes*, VR-RNA in *C. perfringens*, CiaRH in *S. pneumoniae* and LhrC in *L. monocytogenes* are all regulated through a two-component system. RivX is controlled by the CovRS two-component system. CovR is the response regulator whereas CovS is the sensor kinase. CovRS influences a multitude of genes involved in virulence, including the sRNA RivX [73,74]. RivX exhibits positive control over the Mga regulon [11]. VR-RNA is involved in the VirR/VirS two-component system. Genes for virulence factors, several enzymes, and metabolism are controlled by this system [64]. In *S. pneumoniae*, the CiaRH two-component system contains sRNAs referred to as *cia*-dependent small RNAs (csRNAs). These sRNAs are transcribed from five promoters on the CiaRH regulon and have great similarity between each other. This system is heavily involved in competence and virulence in many *Streptococus* species [37,51]. LhrC in *L. monocytogenes* is controlled by the LisRK two-component system. This system controls stress response as well as virulence [72]. Generally, both TCS and sRNAs have multiple target genes. Through the interplay between these multi-target regulators, the signal sensed by the histidine kinase of a TCS can be propagated through diverse sets of genes efficiently to adapt new environment. As more research is performed in gram-positive sRNAs, the interplay between sRNAs and two-component systems will likely be even greater.

### 2.4. Dual Function Riboswitch/sRNA

**Figure 4:**
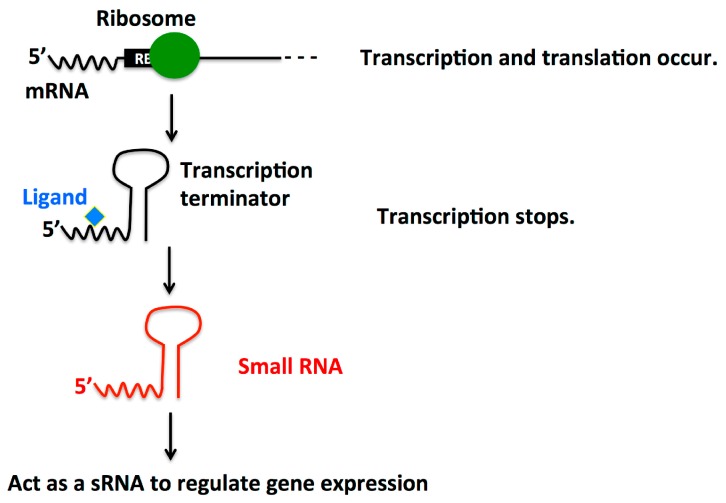
Dual function Riboswitch/sRNA. Riboswitches are regulatory segments of mRNA that can bind a small molecule ligand to induce a structural change. After binding to a ligand, these regulatory segments can form a transcription terminator loop to stop transcription and either be degraded or in some cases, can be utilized as an sRNA. The sRNA then regulates gene expression.

Bacterial riboswitches are present in the 5′-UTR regions of mRNAs and bind ligands to regulate downstream gene expression. Typically, riboswitches regulate the expression of protein-coding genes. However, some results suggest that they can also regulate the expression of noncoding RNAs [75]. Interestingly, a few have been found to act as both a riboswitch and a sRNA (Figure 4).

In *L. monocytogenes*, sRNAs SreA and SreB are each a dual function riboswitch/sRNA. Recently, the sRNA EutX in *E. faecalis* was also found to contain a riboswitch. This riboswitch/sRNA affects the expression of the ethanolamine utilization (*eut*) locus [76]. As more research develops, more dual function riboswitches/sRNAs could be identified.

### 2.5. Small RNAs in Quorum Sensing System

**Figure 5:**
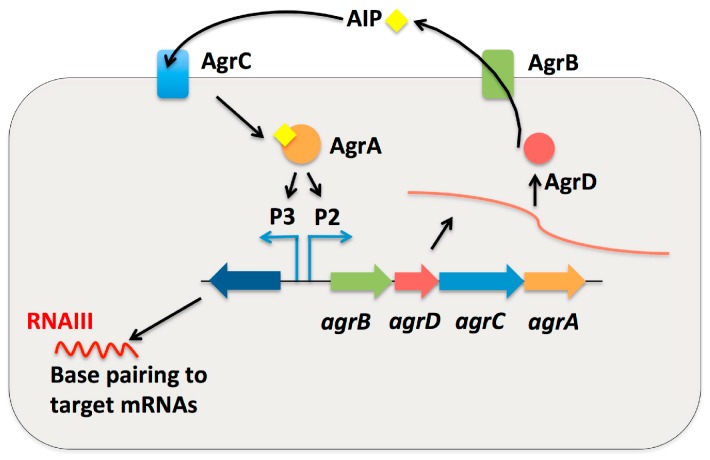
An sRNA is used as an effector molecule in quorum sensing systems. Quorum sensing is a means of cell-cell communication in bacteria to exchange information about cell density and thereby adjust gene expression in the cell. In the *S. aureus* Agr quorum sensing system, the autoinducing peptide (AIP) is synthesized from the AgrD peptide. Following the modification of AgrD, the mature AIP is transported out of the cell through the AIP transporter membrane protein, AgrB. This AIP is then recognized by the two-component system AgrC and AgrA. AgrC is the membrane-bound histidine kinase that is activated by the binding of AIP. AgrA is the response regulator that becomes phosphorylated and this phosphorylated AgrA activates either the P2 or P3 promoter. Activation of P2 regulates the expression of the *agr* operon, also referred to as RNAII, which controls the quorum sensing process. Activation of P3 regulates the expression of the sRNA RNAIII. RNAIII goes on to then regulate the expression of target mRNAs.

Quorum-sensing is a form of communication between bacterial cells through autoinducer molecules. Autoinducers are secreted and then detected by nearby cells after reaching a threshold level. Quorum sensing systems can regulate multiple behaviors such as biofilm formation, bioluminescence, and virulence [77]. Several sRNA-controlled quorum-sensing systems have been studied in gram-negative bacteria such as *V. cholera* and *V. harveyi* [78,79]. Small RNA-controlled quorum-sensing has also been discovered in gram-positive pathogens such as *S. aureus* and *L. monocytogenes.* For sRNA-regulated quorum-sensing in gram-negative bacteria, Hfq is required whereas in gram-positive pathogens Hfq has not been found to be necessary [77]. The *agr* (accessory gene regulation) system is composed of two transcriptional units, RNAII and RNAIII, which are transcribed in opposite directions by P2 and P3 promoters respectively (Figure 5) [80]. RNAII encodes a two-component system involving AgrA and AgrC as well as AgrD, the precursor of the autoinducer, and its export protein AgrB. AgrA influences quorum-sensing through acting as the response regulator whereas AgrC is the sensor histidine kinase [1]. When the autoinducer binds to AgrC, AgrA is then activated and binds to the *agr* promoters to promote gene expression [14,80]. *L. monocytogenes* also utilizes the *agr* system for quorum-sensing control through the AgrD (*lmo0049*) sRNA [25]. Genome analysis of *L. monocytogenes* reveals the same arrangement of the *agr* locus as in *S. aureus* [81]. More studies are necessary to better understand the role of sRNA in the *agr* system of *L. monocytogenes*.

### 2.6. Small RNAs in Toxin/Antitoxin System

Toxin–antitoxin (TA) systems are common in bacteria and serve to benefit the organism’s growth, survival, and pathogenicity. They have been mostly studied in gram-negative bacteria. Each TA system is comprised of both a stable toxin and an easily displaced antitoxin that neutralizes the toxin. There are two main types of TA systems, (1) Antitoxin type I where a sRNA base pairs with the toxin mRNA, preventing protein synthesis; (2) Antitoxin type II where the antitoxin is a protein that binds and inhibits the toxin protein [82]. In either system, if the antitoxin is degraded, the effect of the toxin is no longer inhibited and the cell dies [83]. In *E. faecalis*, the *ef0408-0409* sRNA is the antitoxin, which targets the Fst peptide toxin [32]. A mutant lacking the antitoxin sRNA did not lead to cell death, implying that the system is more complex than originally expected. A computational approach has been used to predict homologs of the *par* (TA) locus in the *S. pneumoniae* chromosome, which discovered the first report of a type I TA system in that pathogen [82]. Known antitoxin sRNAs form secondary structures which can be predicted through computational analysis. More research of TA systems in various gram-positive bacteria could lead to the discovery of shared characteristics and a better understanding of the systems as a whole. Further comparisons of structures could allow for the discovery of new putative type I TA systems in other gram-positive bacteria.

### 2.7. The Involvement of Hfq in sRNA Activity

The Hfq protein is highly conserved and is involved in stabilization of RNAs, including sRNAs [59]. The mechanism by which the Hfq:sRNA interaction facilitates gene regulation is still disputed [59]. In the Hfq remodeling model, Hfq removes structures that would otherwise inhibit sRNA:mRNA duplex formation. Another model predicts Hfq as just a docking platform for the sRNA and the target mRNA. Lastly, Hfq could induce specifically targeted mRNA degradation [23,84,85]. Hfq has been identified as required for *trans*-acting sRNAs in gram-negative bacteria. However, *trans*-acting sRNAs in gram-positive bacteria act independently of Hfq with the exception of LhrA in *L. monocytogenes* [59].

## 3. Conclusions

As more sRNAs in gram-positive bacteria have been studied, various similarities to gram-negative bacteria as well as differences have been discovered. The basic sRNA functions of translation regulation, RNA stabilization, and protein activation are comparable between gram-positive and gram-negative bacteria thus far. Other facets of similarity include the use of two-component systems, quorum sensing, and CU repeated regions.

Two-component systems are widely used throughout bacteria in both gram-positive and gram-negative species. Many sRNAs in gram-positive bacteria are regulated by two-component systems, as discussed previously. Similarly, two-component systems in gram-negative bacteria are used to regulate sRNAs. In *Pseudomonas aeruginosa*, the GacS/GacA TCS controls the sRNAs RsmY and RsmZ that are also involved in quorum sensing [86]. Another example is the BarA/UvrY TCS in *E. coli*, a system that plays a role in biofilm formation, motility, and central carbon metabolism through the regulation of the sRNAs CsrB and CsrC [87]. Communication between cells is an important factor for any bacteria and sRNAs affect this process in both gram-positive and gram-negative bacteria. Two of the best-known gram-negative examples in quorum sensing are *Vibrio cholera* and *Vibrio harveyi.* The mRNA of HapR and LuxR, the quorum sensing regulators in *V. cholera* and *V. harveyi* respectively, are both shown to be destabilized by Hfq, which implies the involvement of sRNAs such as Qrr (Quorum regulatory RNA) [78]. *S. pyogenes*, *S. pneumonia*, *S. aureus*, and *L. monocytogenes* have all been found to employ CU rich motifs for sRNA:mRNA specificity. Recently, a similar motif was discovered in the gram-negative bacteria *Helicobacter pylori.* A CU repeat in the RepG sRNA is used for base-pairing to its target mRNA, *tlpB* [88]. It is likely that as research continues, more CU motifs will be found in both gram-positive and gram-negative bacteria.

The main difference between sRNAs in gram-positives and gram-negatives is the requirement of the chaperone Hfq. Most, if not all, known sRNAs in gram-negative bacteria requires Hfq for their function. On the other hand, most gram-positive sRNAs do not require Hfq. A potential gram-negative specific sRNA mechanism is the binding of sRNA to block a ribosome standby site that thus far has only been documented in *E. coli* [89]*.* Lastly, the dual-function riboswitch/sRNAs have only been identified in the gram-positive pathogens *L. monocytogenes* and *E. faecalis.* However, this discovery is too new to declare it a phenomenon specific to gram-positive bacteria. These similarities and differences regarding sRNAs between gram-positive and gram-negative bacteria can only be viewed as temporary, but there seems to be far more in common than there is different between them. As more research develops in both branches, more similarities and differences are sure to be identified.

An exciting prospect for sRNA research is the possibility for bacterial sRNAs to affect eukaryotic cells [90]. A recent study in the plant pathogen *Agrobacterium tumefaciens* shows sRNAs can be transferred to plant cells through a type IV-secretion system (T4SS). Further research needs to be performed to see if the sRNAs could then interfere with host cell physiology. Some bacterial pathogens such as *Rickettsia* spp. and *H. pylori*, which infect mammalian cells, encode T4SS and evidence for sRNA transfer has been shown [90].

Although there are some unique differences between bacterial sRNAs, many of the foundational characteristics and mechanisms are similar. As such, the study of one helps better the understanding of all through comparative analysis. Because research of sRNAs in gram-positive pathogens is still relatively young, more similarities would be discovered in the future.
